# Listening Through Noise: Robust Ultrasonic Crack Detection in Coal Mine Drill Pipes Using Sliding-Window RMS and CNNs

**DOI:** 10.3390/s26030986

**Published:** 2026-02-03

**Authors:** Xianghui Meng, Hua Luo, Fengli Lei, Xiaoyu Tang, Yongxiang Zhang, Wenbin Huang, Yunfei Xu, Jiaqi Sun, Yinjun Wang

**Affiliations:** 1 State Key Laboratory of Coal Mine Disaster Prevention and Control, CCTEG Chongqing Research Institute, Chongqing 400039, China; 2 State Key Laboratory of Mechanical Transmission for Advanced Equipment, Chongqing University, Chongqing 400044, China; 3 Chongqing Key Laboratory of Green Design and Manufacturing of Intelligent Equipment, Chongqing Technology and Business University, Chongqing 400067, China

**Keywords:** drilling pipes, ultrasonic crack detection, bulk waves, convolutional neural network

## Abstract

Coal mine drill pipes are subjected to periodic impacts and high-intensity loads in complex underground environments, making them prone to developing micro-cracks that gradually expand, leading to equipment failure and major safety accidents. To address this issue, this paper proposes a framework for ultrasonic crack detection in drill pipes, which leverages a sliding-window root mean square (SWRMS) index for feature representation and a convolutional neural network for accurate classification in noisy environments. The influence mechanism of cracks on ultrasonic echoes was studied, and the SWRMS index was introduced to characterize the ultrasonic signal features. This index reflects the spatial position of the crack through the peak position and reveals the crack size through the amplitude, achieving a unified representation of both crack position and size. Furthermore, to address challenges such as spurious echoes and noise interference caused by the drill pipe’s threaded structure in practical engineering applications, convolutional neural network (CNN) was constructed to achieve intelligent identification of drill pipe cracks in high-noise environments. A data augmentation method using alternating noise levels was designed to simulate the scattering effect caused by the drill pipe’s threads and actual noise interference. The results show that CNN exhibits superior recognition performance under different noise levels, maintaining a classification accuracy of 94.4% even at a 75% noise level. The research results verify that the proposed method has significant advantages in crack detection accuracy and noise robustness, providing effective support for real-time monitoring and intelligent diagnosis of key components such as coal mine drill pipes.

## 1. Introduction

Drill pipes serve as a crucial component in underground coal-mine drilling operations [1]. Their performance directly influences the efficiency and safety of coal resource development [2]. A drill pipe generally consists of a pipe body and a joint, which are integrated via friction welding. During drilling, the drill pipe is connected to other drilling tools through the joint’s threads. Owing to the frequent torque transmission and tensile loads that the joint endures [3], its diameter is typically larger than that of the pipe body. Nevertheless, this structural characteristic also gives rise to a significant stress concentration phenomenon at the joint, where the thread root becomes a high-risk area for crack initiation. Once cracks propagate, they not only impede drilling efficiency but can also trigger severe underground accidents, presenting substantial engineering safety hazards.

Currently, on-site engineering for crack detection in coal-mine drill pipes employs methods such as magnetic particle testing, eddy current testing, visual inspection, and ultrasonic testing. Magnetic particle testing is a non-destructive testing approach based on the principle of magnetic field leakage [4]. After magnetizing ferromagnetic materials, surface or near-surface cracks or other defects cause local distortion of magnetic flux lines, resulting in magnetic field leakage [5]. By sprinkling magnetic powder on the defect area, the magnetic powder accumulates at the leakage magnetic field, forming a clearly visible defect indication. Chen et al. [6] proposed a magnetic particle testing method based on 3D contour measurement for the automatic identification of cracks in ferromagnetic materials. This method identifies cracks by detecting contour changes caused by magnetic powder accumulation, obviating the need for visual feature enhancement. Eddy current testing operates on the principle of electromagnetic induction. When an alternating current passes through the probe, it generates an alternating magnetic field. When the probe approaches a conductive material, an eddy current is induced within it [7]. Cracks or other defects in the material cause abnormal local eddy current distribution, leading to changes in the detection signal. Kiselev et al. [8] proposed a method for measuring the inner diameter of conductive pipes using eddy current testing. The visual recognition method is based on image acquisition and computer vision technology [9]. High-resolution camera equipment is used to collect surface images of objects, and techniques such as edge detection and deep learning are combined to identify possible crack or damage features in the images. Francesca et al. [10] proposed and verified a method for detecting surface cracks using visual and tactile perception. Peng et al. [11] used low-frequency L(0,1) ultrasonic guided waves and both linear and nonlinear indices to detect fatigue cracks in threaded steel rods, showing that nonlinear indices are more sensitive to early damage. The proposed algorithm locates cracks in a remote environment by shooting videos/photos with a robot camera. Although these methods have broad application prospects, they still encounter various problems and challenges. Magnetic particle testing is only applicable to ferromagnetic materials and relies on manual judgment, introducing subjectivity. It shares the same detection depth limitation as eddy current testing and cannot detect deep-seated cracks. Visual inspection is also restricted to visible surface cracks and is sensitive to environmental lighting, surface contamination, and oil stains.

In ultrasonic non-destructive testing, surface waves and guided waves are commonly used for defect detection in large-scale structures [12,13,14,15,16,17]. However, given the complex geometric structure and relatively small diameter of coal-mine drill pipes, guided waves are prone to strong scattering by the threaded structure and boundary conditions during propagation, resulting in mode conversion and signal distortion [18,19,20,21,22]. This makes it difficult to ensure the stability and accuracy of crack identification. In contrast, bulk waves have the characteristics of clear propagation paths, concentrated energy, and sensitivity to local defects [23,24,25,26]. Their reflection characteristics can more intuitively reveal the existence and size information of cracks. Moreover, bulk wave signals are less affected by the scattering of the threaded structure, which can enhance the reliability of crack location and quantitative analysis while maintaining detection sensitivity. Therefore, this paper selects bulk waves as the main excitation form for crack detection in drill pipes to fully exploit their advantages in the defect diagnosis of complex structural components. The main contributions of this paper are as follows: (1) A bulk-wave-based ultrasonic detection mechanism for coal-mine drill pipes is established and experimentally verified. It reveals how crack position and size affect the arrival time, amplitude, and waveform characteristics of echo signals. A sliding-window root mean square (SWRMS) index is proposed, which uses the peak position to indicate crack location and the peak amplitude and width to characterize crack size, providing a unified feature representation for both. (2) A convolutional neural network (CNN)-based intelligent recognition framework is constructed. One-dimensional convolutions, ReLU activation, max pooling, and global average pooling are utilized to automatically extract crack-related features from noisy ultrasonic signals. A noise-aware data augmentation strategy that alternately superimposes multi-level Gaussian noise to simulate thread scattering and field interference is combined. Experimental results on this multi-noise dataset demonstrate strong robustness, with the network maintaining high crack identification accuracy (up to 94.4% even at a 75% noise level), thereby verifying the practical effectiveness of the proposed method for real-time monitoring and intelligent diagnosis of drill pipes.

The remainder of this paper is structured as follows. Section 2 elaborates on the complete method, from theoretical principles and feature extraction to network architecture and experimental settings. Section 3 presents comprehensive experimental results validating the proposed method. Section 4 discusses the significance and limitations of the research and summarizes the work.

## 2. Methodology

### 2.1. Principle of Drilling Pipe Crack Detection Based on Ultrasonic Bulk Waves

In ultrasonic non-destructive testing, a bulk wave is an elastic wave that propagates within a solid medium. It primarily consists of two basic forms: the longitudinal wave and the transverse wave. In a longitudinal wave, the particle vibration is parallel to the wave propagation direction; it is a form of wave dominated by volumetric strain and has a faster propagation speed. The transverse wave is a shear wave where the particle vibration is perpendicular to the propagation direction; it has a slower propagation speed but is more sensitive to discontinuities within the material. Since coal mine drill pipes are typically made of isotropic steel, the propagation of ultrasonic waves within them can be described by linear elastic wave theory. The propagation of bulk waves in isotropic elastic solids follows the Navier equation, which is the fundamental governing equation describing the relationship between particle motion, stress, and strain in elastic media [27]:

(1) $$\left(\lambda +\mu \right)\nabla \left(\nabla \cdot u\right)+\mu \nabla^{2}u=\rho \frac{\partial^{2}u}{\partial t^{2}}$$

where *u* is the displacement vector of the particle and *ρ* is the medium density. *λ* and *μ* are the Navier constants, which are determined by the elastic properties of the material. ∇ is the gradient operator, ∇*u* is the displacement gradient, and ∇^2^ is the Laplacian operator. This equation can be decomposed into two independent equations, corresponding to the longitudinal wave velocity and the transverse wave velocity:

(2) $$v_{p}=\sqrt{\frac{\lambda +2\mu }{\rho }}$$

(3) $$v_{s}=\sqrt{\frac{\mu }{\rho }}$$

When an ultrasonic wave encounters an interface between two different media, part of its energy is reflected back into the original medium, while the remaining part is transmitted through the interface. This phenomenon is governed by the acoustic impedance Z (Z = *ρc* where *ρ* is the density and *c* is the wave velocity). The acoustic pressure reflection coefficient (*R_p_*) and transmission coefficient (*T_p_*) are given by [28]:

(4) $$R_{p}=\frac{Z_{2}-Z_{1}}{Z_{2}+Z_{1}}$$

(5) $$T_{p}=\frac{2Z_{2}}{Z_{2}+Z_{1}}$$

**Figure 1:**
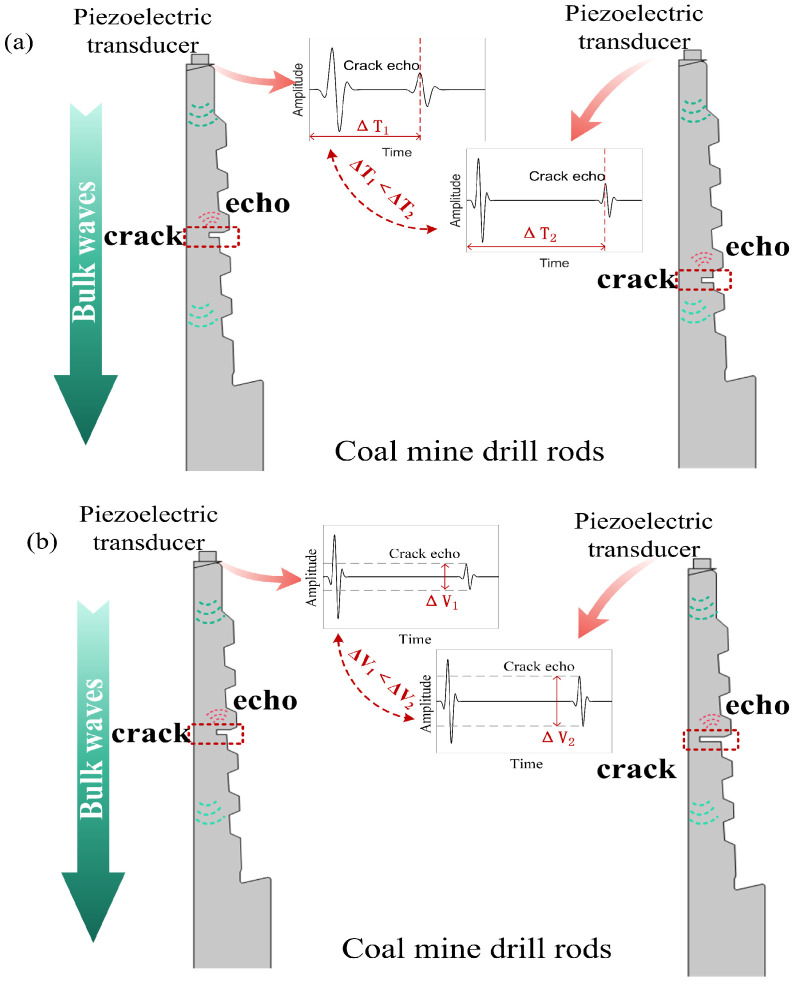
Principle of crack detection in drill pipes. (**a**) Physical mechanisms of the position of the crack affecting bulk wave propagation. (**b**) Physical mechanisms of the size of the crack affecting bulk wave propagation.

In coal mine drill pipes, the propagation behavior of ultrasonic bulk waves is highly sensitive to the material’s structural continuity. In the absence of cracks, the bulk waves emitted from the excitation end propagate at a stable speed within the material, forming regular reflections upon encountering geometric boundaries such as the bottom or threads. However, when bulk waves encounter an internal crack, a significant response occurs. A crack represents a geometric discontinuity, causing an abrupt change in the material’s acoustic impedance. This impedance mismatch leads to the reflection, diffraction, and mode conversion of ultrasonic waves. These phenomena introduce new reflection signals, causing distortion of the overall waveform, asymmetric features in the time domain, and a shift in the energy distribution in the frequency domain. As shown in Figure 1a, when the crack is located close to the position where the excitation probe is arranged, the ultrasonic wave encounters the interface discontinuity within a very short propagation distance after entering the material. The closer the crack is to the probe, the shorter the time required for the reflected wave to return to the receiving end, and thus it appears as a high-amplitude signal that emerges early in the echo signal. In contrast, when the crack is located in the middle of the drill pipe or near the bottom surface, the incident wave needs to propagate a longer distance within the material before reaching the crack position. Therefore, its reflected signal is significantly delayed in the time domain, and the echo arrival time is later.

Furthermore, the difference in crack length will directly affect its ability to reflect ultrasonic waves and influence the characteristics of the echo signal. As shown in Figure 1b, when the crack is short, due to its limited extension range in the material, the area that can effectively interact with the ultrasonic beam is relatively small, resulting in a weaker reflection signal, a lower echo amplitude, and an unclear waveform profile. As the crack length increases, the effective area of interaction with the ultrasonic wave also expands accordingly, allowing for the reflection of more incident energy. At this time, the echo amplitude in the received signal significantly increases, and the signal shape becomes clearer and more stable. These propagation changes constitute the physical basis for crack detection. By analyzing the arrival time, amplitude changes, and waveform distortion of the signal, the existence, location, and size of the crack can be determined.

### 2.2. Drill Pipe Crack Detection Feature Index Based on Sliding-Window Root Mean Square

In ultrasonic testing, the presence of cracks alters the propagation path of sound waves within the material. When ultrasonic waves propagate through the drill pipe and encounter cracks, the sudden change in acoustic impedance at the crack site causes some of the energy to be reflected back to the receiving end. This reflected signal typically does not manifest as a single sharp pulse but rather as a group of echoes with enhanced amplitude and expanded waveform. During this period, the signal energy in the region with cracks is significantly higher than that in the areas without cracks, resulting in a distinct energy peak. The position of this energy peak corresponds to the arrival time of the crack reflection in the original echo signal, thus enabling the estimation of the location of the crack. While its peak height and width reflect the strength and duration of the echo signal, which are related to the size characteristics of the crack.

The root mean square (RMS) value is a commonly used amplitude characteristic index, which is often employed to describe the energy distribution of a signal within a certain time interval:

(6) $$\mathrm{RMS}=\sqrt{\frac{1}{N}\sum_{i=1}^{N}{v_{i}}^{2}}$$

where *v_i_* represents the voltage signal of the ultrasonic probe, and *N* is the signal length. In order to describe this energy enhancement phenomenon more accurately and continuously, this paper introduces the RMS method based on sliding-window calculation, i.e., SWRMS. This method calculates the RMS of the local signal at each moment by applying a fixed-length sliding time window to the ultrasonic signal and obtains an energy envelope curve that changes with time. The value of RMS for the *m*-th window is:

(7) $$\mathrm{SWRMS}=\sqrt{\frac{1}{N}\sum_{i=0}^{N-1}v^{2}\left(mS+i\right)}$$

where v is the voltage signal of the ultrasonic probe, *N* is the window length, and *S* is the sliding step size. In ultrasonic crack detection, commonly used signal metrics include RMS, peak factor, entropy-based measures, and time–frequency features such as wavelet coefficients. These metrics are mainly designed to characterize the overall amplitude distribution or complexity of the signal, and they are often used as discriminative features for damage classification. However, most of these metrics provide limited direct information about the temporal localization of ultrasonic echoes. Compared with those metrics, SWRMS can not only accurately reflect the position of the crack reflection wave on the time axis but also evaluate the crack size characteristics through the peak size and wave packet width, thereby achieving the dynamic perception of the echo amplitude and energy distribution and effectively characterizing the local reflection enhancement phenomenon caused by the crack. In this paper, the window length is selected as 40 sampling points, and the step size is 15 sampling points. To avoid the influence of the excitation signal on the results, the starting point of the calculation is the 200th sampling point.

### 2.3. CNN-Based Drill Pipe Crack Identification

#### 2.3.1. The Method of CNN-Based Drill Pipe Crack Identification

In the detection of cracks in coal mine drill pipes, the threaded structure and complex boundary conditions introduce strong background noise and multiple scattering components into the ultrasonic echoes. This interference makes traditional recognition methods based on fixed thresholds or features unreliable. To overcome this, this paper further introduces a CNN to classify and discriminate the ultrasonic detection signals of drill pipes. The convolution kernel of the CNN is essentially a learnable band-pass filter, which can adaptively suppress noise and clutter unrelated to the cracks during training and amplify the transient features related to the cracks [29]. This inherent robustness makes CNN particularly suitable for the processing and intelligent recognition of ultrasonic signals of drill pipe cracks in noisy environments.

**Figure 2:**
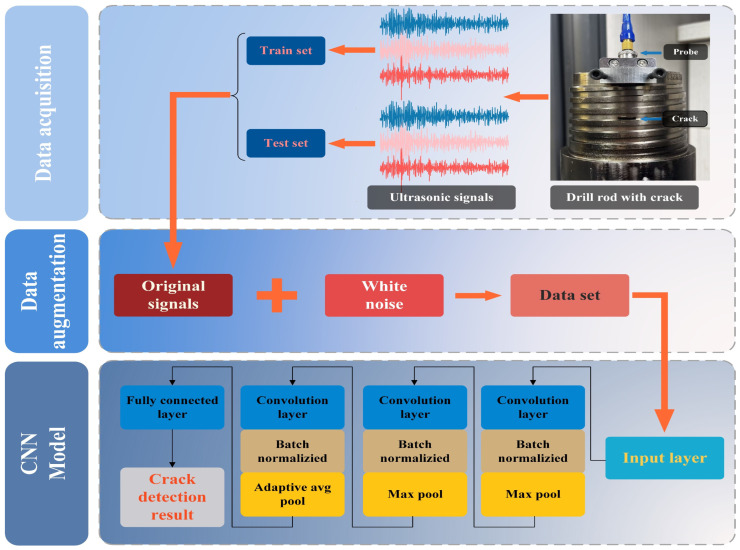
The framework of CNN-based drill pipe crack identification.

Figure 2 illustrates the overall framework of the proposed CNN-based drill pipe crack identification method. First, ultrasonic echo signals are acquired from a drill pipe specimen with and without cracks. These raw signals are labeled and divided into a training set and a test set. To improve robustness to field interference, a data augmentation stage is then applied, in which white noise of different intensities is superimposed on the original signals, forming an expanded ultrasonic dataset that better reflects the complex, noisy environment of coal mine drilling. This augmented dataset is subsequently fed into a one-dimensional CNN model. At the input layer, each ultrasonic signal (or its corresponding feature sequence) is taken as a one-dimensional time series. The signal then passes through several convolutional blocks, where each block consists of a convolution layer, batch normalization, a nonlinear activation, and max-pooling. These operations automatically extract local crack-related features, suppress irrelevant noise, and gradually reduce the temporal dimension. After the convolutional stages, an adaptive (global) average pooling layer aggregates the high-level feature maps into a compact feature vector, which is finally passed to a fully connected layer and a Softmax classifier. The network outputs the crack detection result, i.e., the predicted crack category of the drill pipe.

The derivation of the proposed method is as follows:

(1) The one-dimensional convolution calculation formula can be expressed as:

(8) $${y_{i}}^{\left(k\right)}=b^{\left(k\right)}+\sum_{c=1}^{C_{m}}\sum_{m=0}^{M-1}w_{c,m}^{\left(k\right)}\cdot x_{i\cdot s+m}^{\left(c\right)}$$

where *x* is the input signal, $w_{c,m}^{\left(k\right)}$ denotes the *m*-th weight of the convolution kernel on the *k*-th channel, *M* is the convolution kernel length, *s* is the stride, *b*^(*k*)^ is the bias term, and $y_{i}^{\left(k\right)}$ is the output of the *k*-th feature channel at position *i* after convolution. Through this local weighted summation and weight sharing mechanism, the network can automatically capture the short-term energy enhancement and structural changes in the ultrasonic detection signals of the drill pipe, which are closely related to the size of the cracks. Thus, the size of the cracks can be identified and judged.

(2) The convolution results then need to be transformed through a nonlinear activation function to enhance the model’s expressive power and avoid simple linear mapping. In this paper, the rectified linear unit (ReLU) is adopted. This pointwise nonlinear operation can effectively highlight the mutated parts of the signal, enabling the model to remain sensitive to crack features even when faced with complex noise. Its expression is:

(9) f(z)=max(0,z)

(3) As the convolutional layers are continuously stacked, the network can gradually acquire a wider receptive field. However, it still needs to compress the time dimension to reduce the data size. To achieve this, the maximum pooling operation is used to downsample the features. This operation can extract the most significant response of the signal within a certain time window, thereby retaining key features such as crack reflections, while weakening the influence of irregular noise. Its expression is:

(10) $$y[i]=\underset{j=0,\ldots ,P-1}{\max}x[i\cdot p+j]$$

where *x* is the input signal, *P* is the pooling stride, *P* is the pooling window size, and *y*[*i*] is the output value of the *i*-th pooling operation.

(4) At the network’s end, global average pooling (GAP) is utilized to compress the temporal dimension features into a single global feature vector. Eventually, the global features output by GAP are sent to the fully connected layer and the Softmax classifier, resulting in the probability distribution of crack categories. The calculation formula is as follows:

(11) $$\hat{p_{c}}=\frac{\exp(z_{c})}{\sum_{j}\exp(z_{j})}$$

where *z_c_* indicates the output of the fully connected layer for the *c*-th class, and $\hat{p_{c}}$ represents the probability of predicting that it belongs to this class.

The training objective of the network is achieved through the cross-entropy loss function, which can be calculated as:

(12) $$\Gamma_{CE}=-\sum_{c}y_{c}\log{\hat{p}}_{c}$$

By minimizing this loss, the network can continuously adjust its parameters and improve the accuracy of crack detection.

#### 2.3.2. Dataset Construction

In terms of dataset construction, each crack position was subjected to 12 repeated detections to simulate the multiple measurement scenarios that may exist in the actual engineering detection environment, thereby enhancing the representativeness and reliability of the data. Six of the collected data were used as a training set, and the other six as a test set to ensure the independence and generalization of the model evaluation. Each group of signals contained three types of typical samples: one without a crack signal and two signals with different crack depths (1.5 mm and 4 mm), representing the ultrasonic echo characteristics under different defect conditions. It should be noted that the present dataset is designed for feasibility verification under limited experimental conditions rather than for exhaustive coverage of all possible crack scenarios.

To simulate the complex environment faced by coal mine drill pipes in actual detection, especially the strong background noise and scattering effect caused by the drill pipes’ surface threads and irregular structure, this paper alternately superimposes different intensities of Gaussian white noise on the original signals. Specifically, based on the RMS intensity of the signal, Gaussian white noise with 25%, 50%, and 75% intensities was added, respectively, so that three noise contamination samples could be obtained under each crack condition. Each type of sample was ultimately expanded to 6 enhanced samples, and the test set and training set each contained 18 different signals of the three types of typical samples, thus forming a complete multi-noise level dataset. That is to say, the dataset can be divided into a noise training group (the training set adds different degrees of noise, and the test set remains unchanged) and a noise test group (the test set adds different degrees of noise, and the training set remains unchanged). In this way, the sample quantity can be significantly expanded under the limited experimental collection conditions, alleviating the problem of data scarcity; more importantly, it can effectively simulate the noise environment of the drill pipe in actual service conditions, exposing the model to multiple noise scenarios during training, thereby learning the ability to extract crack echo features from noisy signals.

## 3. Verification and Results

### 3.1. Experimental Setup

**Figure 3:**
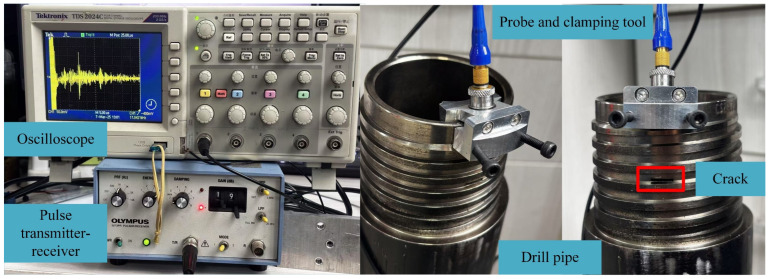
Experimental setup of Drill Pipe.

To validate the effectiveness of the proposed crack detection method, an experimental ultrasonic testing system is constructed, as shown in Figure 3. The system comprised an Olympus 5073PR ultrasonic pulser–receiver (Olympus Corporation, Tokyo, Japan), an Olympus C542-SM 2.25 MHz (Olympus Corporation, Tokyo, Japan) angled beam ultrasonic probe, a water-based couplant, a Tektronix TDS 2024C oscilloscope (Tektronix, Beaverton, OR, USA), and a probe clamping fixture, the test data are shown in Table 1.

The drill pipe samples used in the experiment were 4137H steel hollow male connectors, with a length of 500 mm and a diameter of 89 mm. The material had a uniform structure and no obvious initial defects. The crack simulation was carried out using the mechanical processing method to prepare U-shaped slots of different depths to replace the acoustic characteristics of the real cracks. To avoid the influence of cracks being too close to each other on the detection signals between them, two cracks were artificially machined on the circumference of the drill pipe, with an angle of 120° between the cracks. At the same time, ultrasonic echo signals were collected in the non-crack area as a reference benchmark for comparison with the signals containing cracks. The distance between the two cracks from the end face was 34 mm, the crack width was 1 mm, the crack length was 10 mm, and the depths were 1.5 mm and 4 mm, respectively. To ensure that the ultrasonic waves could effectively enter the interior of the drill pipe and reduce interface losses, a water-based polymer coupling agent was used to improve the energy coupling efficiency. The probe was fixed on the threaded end face of the drill pipe using a clamping fixture and maintained good contact with the end face to approximately simulate the boundary conditions of the piezoelectric excitation source in the simulation model. The pulser–receiver generated the excitation signal, and the oscilloscope collected the echo signals at a sampling rate of 50 MHz.

### 3.2. Performance Verification of SWRMS

#### 3.2.1. Crack Location Detection Results

**Figure 4:**
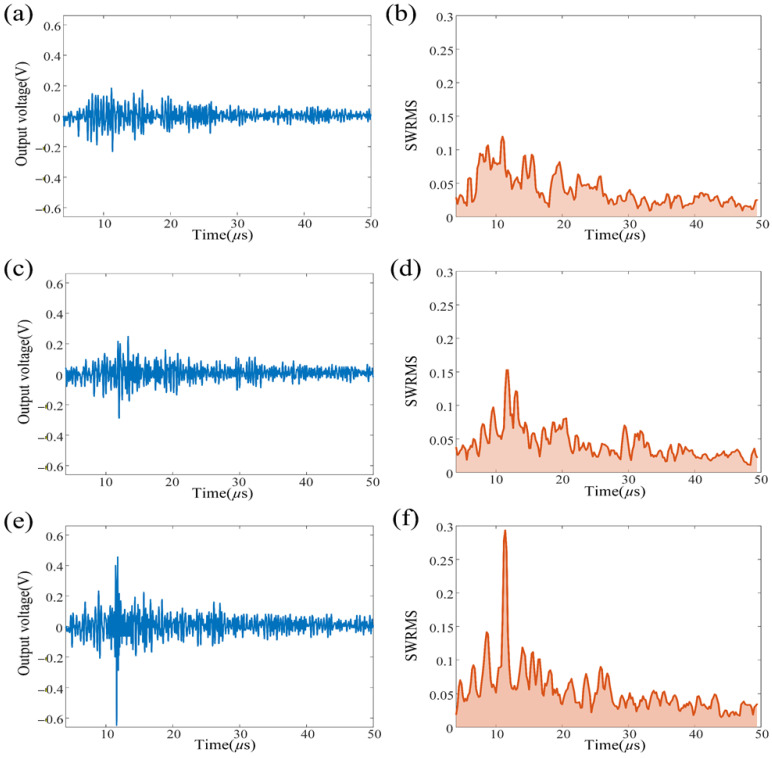
Experimental results of drilling pipe crack location detection. (**a**) Time-domain signal of ultrasonic bulk waves without crack; (**b**) SWRMS without crack; (**c**) Time-domain signal of ultrasonic bulk waves with 1.5 mm crack; (**d**) SWRMS with 1.5 mm crack; (**e**) Time-domain signal of ultrasonic bulk waves with 4 mm crack; (**f**) SWRMS with 4 mm crack.

To validate the proposed index, ultrasonic signals from different areas were processed using the SWRMS method (Equations (6) and (7)). The results are presented in Figure 4.

The time-domain signal from the crack-free area exhibits some amplitude response due to scattering from the surface threads, but no distinct abnormal echoes are observed. In contrast, signals from the 1.5 mm and 4 mm crack areas show significant differences. New echo components corresponding to the crack reflection are superimposed on the signal. After applying the SWRMS calculation, these differences become even more apparent. Clear peaks emerge on the SWRMS curves for the cracked samples. By identifying the time corresponding to these peaks, the ultrasonic wave’s time-of-flight can be determined, and thus the spatial position of the crack can be calculated. The results, shown in Table 2, indicate that the calculated crack positions are highly consistent with the actual positions, with a detection error of less than 1.5%. This verifies the effectiveness of the proposed index for crack localization.

#### 3.2.2. Crack Size Detection Results

The influence of crack size on signal characteristics was analyzed in both the frequency and time domains. A Fast Fourier Transform was applied to the signals, with the analysis focused on the probe’s 6 dB effective bandwidth (1.39–3.72 MHz).

**Figure 5:**
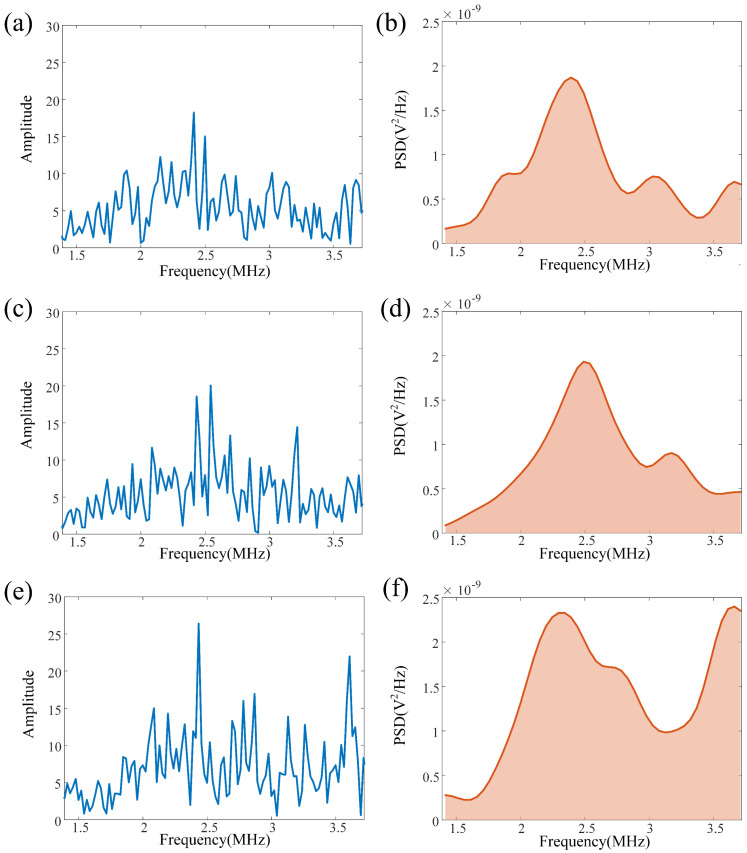
Experimental results of drilling pipe crack size detection. (**a**) Spectrum peak of ultrasonic bulk waves without crack; (**b**) Power spectral density without crack; (**c**) Spectrum peak of ultrasonic bulk waves with 1.5 mm crack; (**d**) Power spectral density with 1.5 mm crack; (**e**) Spectrum peak of ultrasonic bulk waves with 4 mm crack; (**f**) Power spectral density with 4 mm crack.

The results show (Figure 5) that as the crack size increases, the spectral amplitude of the signal within the effective bandwidth shows a gradually increasing trend: the spectral amplitude of the sample without cracks is the lowest, the amplitude corresponding to the 1.5 mm crack increases somewhat, and the 4 mm crack reaches the highest level. Further calculation of the power spectral density (PSD) of the signal also shows a change pattern of overall amplitude enhancement with the increase in crack size, as shown in Table 3.

This trend is also reflected in the time-domain SWRMS characteristics (Figure 4). It can be observed that the peak amplitude of the reflection corresponding to the crack increases gradually with the increase in crack length. For the 1.5 mm crack, the reflection energy is limited, and only a relatively weak local peak is generated on the RMS curve. When the crack extends to 4 mm, the reflection energy significantly increases, and the peak amplitude is significantly higher than the former.

This phenomenon occurs because a larger crack presents a larger reflective surface and a greater acoustic impedance discontinuity, causing more ultrasonic energy to be reflected back to the probe. This is manifested as a concentration of energy and a significant increase in amplitude. Both frequency-domain and time-domain analyses show a consistent trend, further validating the effectiveness of the proposed SWRMS index for identifying crack size.

### 3.3. Performance of Crack Identification in Strong-Noise Environments

Given the challenges of complex noise and signal aliasing in engineering applications, a CNN was introduced for robust classification. To evaluate its performance, the CNN model was tested under the two different dataset scenarios described in Section 2.3.2 (noise training group and noise test group) and compared against a Multi-layer perceptron (MLP) and a Long short-term memory (LSTM) network. Although more advanced architectures, such as Transformer- and autoencoder-based models, have been reported in recent studies, MLP and LSTM were adopted in this work as representative lightweight baselines due to their proven effectiveness, lower computational overhead, and suitability for real-time industrial applications. The MLP consists of three fully connected layers with 512 and 256 hidden neurons, respectively. ReLU activation functions are applied after each hidden layer, and dropout with a rate of 0.3 is used for regularization to mitigate overfitting. The input dimension of the MLP is set to 2300, corresponding to the length of the ultrasonic signal, and the output layer produces three class probabilities. The LSTM model is designed to capture temporal dependencies in the ultrasonic signals. It employs a single-layer bidirectional LSTM with 64 hidden units. The output of the final time step is fed into a fully connected classifier consisting of a 64-neuron hidden layer with ReLU activation and a dropout rate of 0.3, followed by the output layer. This configuration balances representational capacity and computational efficiency for industrial applications. Labels 0, 1, and 2 correspond to crack sizes of 0 mm, 1.5 mm, and 4 mm, respectively.

**Figure 6:**
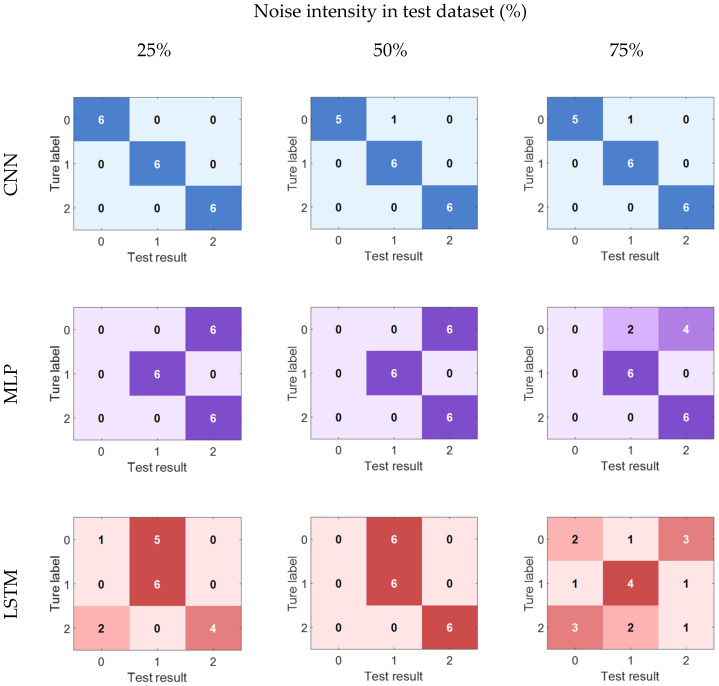
Confusion matrix of different models under different noise intensities in test dataset.

In the noise test group experiments, the training set is the original signal, and the test set is overlaid with different intensities of Gaussian white noise. At this time, the performance differences of various networks are further amplified, as shown in Figure 6. When 25% noise is added to the test set, the accuracy rates of MLP and LSTM are 66.7% and 61.1%, respectively, while CNN still remains at 100%, demonstrating a significant advantage. Under the most severe 75% noise condition, CNN can still achieve a recognition rate of 94.4%, while LSTM and MLP are only 66.7% and 38.9%, respectively. This result fully demonstrates that when the model needs to perform crack recognition in an unseen noise environment, the generalization ability of CNN is significantly superior to traditional MLP and time-series modeling LSTM.

**Figure 7:**
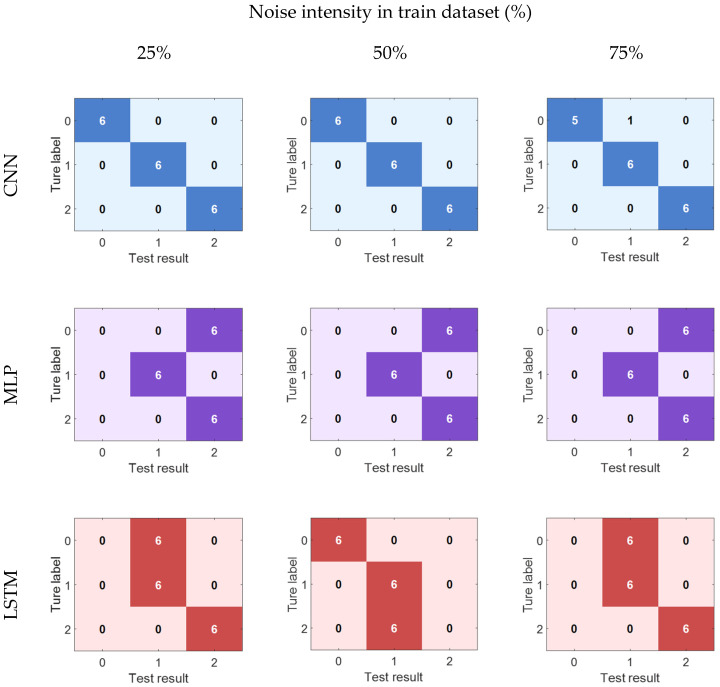
Confusion matrix of different models under different noise intensities in train dataset.

In the noise training group experiment, the training set was overlaid with different intensities of Gaussian white noise, while the test set remained with the original signal unchanged. The overall results indicated that as the noise intensity increased, the classification performance of each model decreased, but the decline did not occur in the same way, as shown in Figure 7. For MLP and LSTM, the introduction of noise not only led to a numerical decrease in classification accuracy but also to a more significant phenomenon: the confusion in class distinction of the models intensified. For example, when the noise intensity reached 25% RMS, the recognition accuracy of 0 mm crack signals by MLP significantly decreased, and a large number of samples were misjudged as 1.5 mm cracks; LSTM also had a similar problem under the condition of 50% noise: one-third of the 4 mm crack samples were wrongly identified as 1.5 mm cracks. In contrast, the performance of CNN was more stable: even under the condition of 75% RMS strong noise, it could still maintain a 100% overall recognition rate.

## 4. Conclusions

In this study, an ultrasonic bulk-wave-based framework for crack detection in coal mine drill pipes was proposed and systematically validated. By analyzing the propagation behavior of bulk waves in intact and damaged drill pipes, the physical mechanisms linking crack position and crack size to the arrival time, amplitude, and waveform distortion of ultrasonic echoes were clarified. On this basis, a sliding-window root mean square (SWRMS) index was introduced to construct an energy envelope of the echo signal. The peak position of the SWRMS curve effectively indicates the spatial location of the crack, while the peak amplitude and width reflect the severity of the damage. In this way, the SWRMS feature realizes a unified representation of both crack position and crack size and provides a clear, interpretable basis for subsequent intelligent identification. To cope with the strong noise and complex scattering effects caused by the threaded joints and harsh underground conditions, a CNN-based crack identification model was further designed. The one-dimensional CNN, incorporating convolution, batch normalization, ReLU activation, max pooling, and global average pooling, can automatically learn discriminative features from ultrasonic signals and suppress spurious echoes and random noise. A multi-noise-level 5dtzxd dataset was constructed by repeatedly measuring typical defect states (no crack, 1.5 mm crack, and 4 mm crack) and superimposing Gaussian white noise with different intensities, thereby simulating practical detection environments. Experimental results show that the proposed method maintains high recognition performance under different noise levels, achieving an accuracy of up to 94.4% even at a 75% noise level, which verifies its strong robustness and engineering applicability for real-time monitoring and intelligent diagnosis of drill pipes. Although the proposed framework demonstrates promising performance, its generalization beyond the tested scenarios needs further validation with larger and more varied datasets. Future work will focus on expanding the range of crack types and geometric configurations, investigating real-time diagnostic performance, including inference speed optimization and deployment on embedded or industrial platforms, integrating more complex loading and environmental conditions, and combining additional sensing modalities or advanced network architectures to further enhance the generalization ability and real-time performance of the system. These efforts are expected to promote the practical deployment of intelligent ultrasonic inspection for key components in coal mine drilling equipment.

## Figures and Tables

**Table 1:** Dataset composition under different noise conditions.

Crack Condition	Original Signals	Noise Levels	Training Samples	Test Samples
No crack	12	3	18	18
Crack 1.5 mm	12	3	18	18
Crack 4 mm	12	3	18	18

**Table 2:** Result of crack location detection using SWRMS.

Crack Size	Distance to Top (mm)		Error (%)
	Actual Distance	Detected Distance	
Crack 1.5 mm	34.00	34.50	1.47
Crack 4 mm	34.00	33.63	1.09

**Table 3:** Spectral characteristics of different crack sizes.

Crack Size	Spectrum Peak	Power Spectral Density (V^2^/Hz)
Without crack	18.249	1.8094 × 10^−3^
Crack 1.5 mm	20.048	1.9245 × 10^−3^
Crack 4 mm	26.400	3.2074 × 10^−3^

## Data Availability

The original contributions presented in this study are included in the article. Further inquiries can be directed to the corresponding author.
